# Impact of targeting the platelet-activating factor and its receptor in cancer treatment

**DOI:** 10.1186/s40779-025-00597-0

**Published:** 2025-03-04

**Authors:** Kimya Qaderi, Arvin Shahmoradi, Anita Thyagarajan, Ravi P. Sahu

**Affiliations:** 1 Department of Molecular and Cell Biology, College of Life Sciences, University of Leicester, University Road, Leicester, LE1 7RH UK; 2 Department of Laboratory Medicine, Faculty of Paramedical, Kurdistan University of Medical Sciences, Sanandaj, 66177-13446 Kurdistan Iran; 3 Department of Pharmacology and Toxicology, Boonshoft School of Medicine Wright State University, Dayton, OH 45435 USA

**Keywords:** Platelet-activating factor (PAF), Platelet-activating factor-receptor (PAFR), Antagonists, Inhibitors, Cancer, Cell signaling pathways, Radiation therapy, Chemotherapy

## Abstract

The lipid mediator platelet-activating factor (PAF) and its receptor (PAFR) signaling play critical roles in a wide range of physiological and pathophysiological conditions, including cancer growth and metastasis. The ability of PAFR to interact with other oncogenic signaling cascades makes it a promising target for cancer treatment. Moreover, numerous natural and synthetic compounds, characterized by diverse pharmacological activities such as anti-inflammatory and anti-tumor effects, have been explored for their potential as PAF and PAFR antagonists. In this review, we provide comprehensive evidence regarding the PAF/PAFR signaling pathway, highlighting the effectiveness of various classes of PAF and PAFR inhibitors and antagonists across multiple cancer models. Notably, the synergistic effects of PAF and PAFR antagonists in enhancing the efficacy of chemotherapy and radiation therapy in several experimental cancer models are also discussed. Overall, the synthesis of literature review indicates that targeting the PAF/PAFR axis represents a promising approach for cancer treatment and also exerts synergy with chemotherapy and radiation therapy.

## Background

Cancer is a disease characterized by the uncontrolled growth and invasion of aberrant cells, which poses a significant global public health challenge and ranks as the second leading cause of death in the United States [1]. There is a projected surge in the rates of cancer cases and deaths in the coming decades. By the year 2024, it is estimated that there will be approximately 2,001,140 new cases of cancer and 611,720 cancer-related deaths in the United States [1, 2]. The risk factors for cancer are closely linked to the consequences of population expansion and societal progress [3]. Primary prevention, often referred to as ‘lifestyle’, is undoubtedly the most cost-effective strategy for mitigating a considerable portion of the global burden of chronic diseases, including cancer, through behavioral and environmental interventions, as emphasized by the World Health Organization (WHO). According to the WHO study, at least 35% of cancer deaths worldwide can be attributed to modifiable lifestyle factors such as smoking and alcohol consumption in both high-income and low to middle-income countries [4].

The primary objective of cancer treatment is to achieve complete remission, with a secondary focus on palliative care, which aims to prolong life and alleviate pain, in cases where complete remission is not feasible due to advanced disease [5, 6]. Despite significant advancements in oncology, the fact that over 9 million individuals continue to die from cancer annually underscores the limitations of current therapeutic approaches in achieving a definitive cure. Novel methodologies, including pharmaceutical agents, biological therapies, and immune-based interventions, are being increasingly employed for therapeutic purposes. Currently, the main categories of systemic therapies for cancer encompass chemotherapy, immunotherapy, endocrine therapy, targeted therapy, and a combination of these therapies, including chemoradiation [7–10]. Chemotherapy impedes cell proliferation and tumor propagation by selectively targeting rapidly dividing cells, predominantly cancerous cells, which exhibit the highest proliferation rates, thereby preventing invasion and metastasis [8, 9]. However, neutropenia, infection, mucositis, and diarrhea were prevalent side effects of chemotherapy due to the vulnerability of bone marrow and mucosal cells [8]. To mitigate these chemotherapy-associated adverse effects, and address the impact of genetic abnormalities, such as aberrantly expressed oncogenic proteins that drive the proliferation of specific cancer types, monoclonal antibodies, and small-molecule inhibitors are predominantly utilized in targeted therapy [7, 10]. Despite these advancements, cancer cells frequently develop resistance to these therapeutic agents, indicating the need to investigate counteracting mechanisms that inhibit the efficacy of cancer therapies. In this context, the impact and significance of lipid mediators, particularly platelet-activating factor (PAF) and PAF-receptor (PAFR) signaling, have been explored in various disease pathophysiologies, including cancer.

The ability of PAF/PAFR signaling to elicit robust systemic pro-inflammatory, pro-proliferative, and delayed immune-suppressive responses, which are implicated in numerous clinical situations, justified its research in cancer development. This is particularly relevant as many malignant cells have been identified to express PAFR [11–15]. PAF exerts its effects via binding to a seven-transmembrane G-protein-coupled receptor known as the PAFR, which is expressed in diverse cell types, including tumor cells [12, 16–21]. Although the specific configuration of the PAFR binding site remains unknown, it is widely recognized as a therapeutic target, with a variety of compounds possessing different structures being discovered and explored as potential PAF inhibitors and PAFR antagonists [22]. These compounds encompass molecules that mimic the structure of the natural PAF ligand, synthetic heterocycles, intricate polycyclic natural products, and diverse metal complexes [22]. Given that most chemotherapeutic agents and radiotherapy generate PAF agonists and enhance PAFR expression in tumor cells, resulting in tumor cell repopulation, PAF inhibitors or PAFR antagonists have been shown to significantly decrease tumor growth or repopulation [19, 23, 24]. Notably, PAFR antagonists have emerged as a promising approach to enhance the efficacy of chemotherapy and radiation therapy for cancer treatment [23–25].

This narrative review provides a comprehensive summary of the current findings on the structure, activities, and roles of PAF and PAFR. Additionally, it encompasses an in-depth examination of various classes of compounds that target PAF and PAFR. Moreover, this resource not only systematically categorizes the roles of PAF/PAFR signaling in different cancer types but also offers detailed information on the significance of PAF inhibitors and PAFR antagonists in cancer treatment, as well as their impact on the efficacy of radiotherapy and chemotherapeutic agents.

## Structure and activity of PAF

**Fig. 1:**
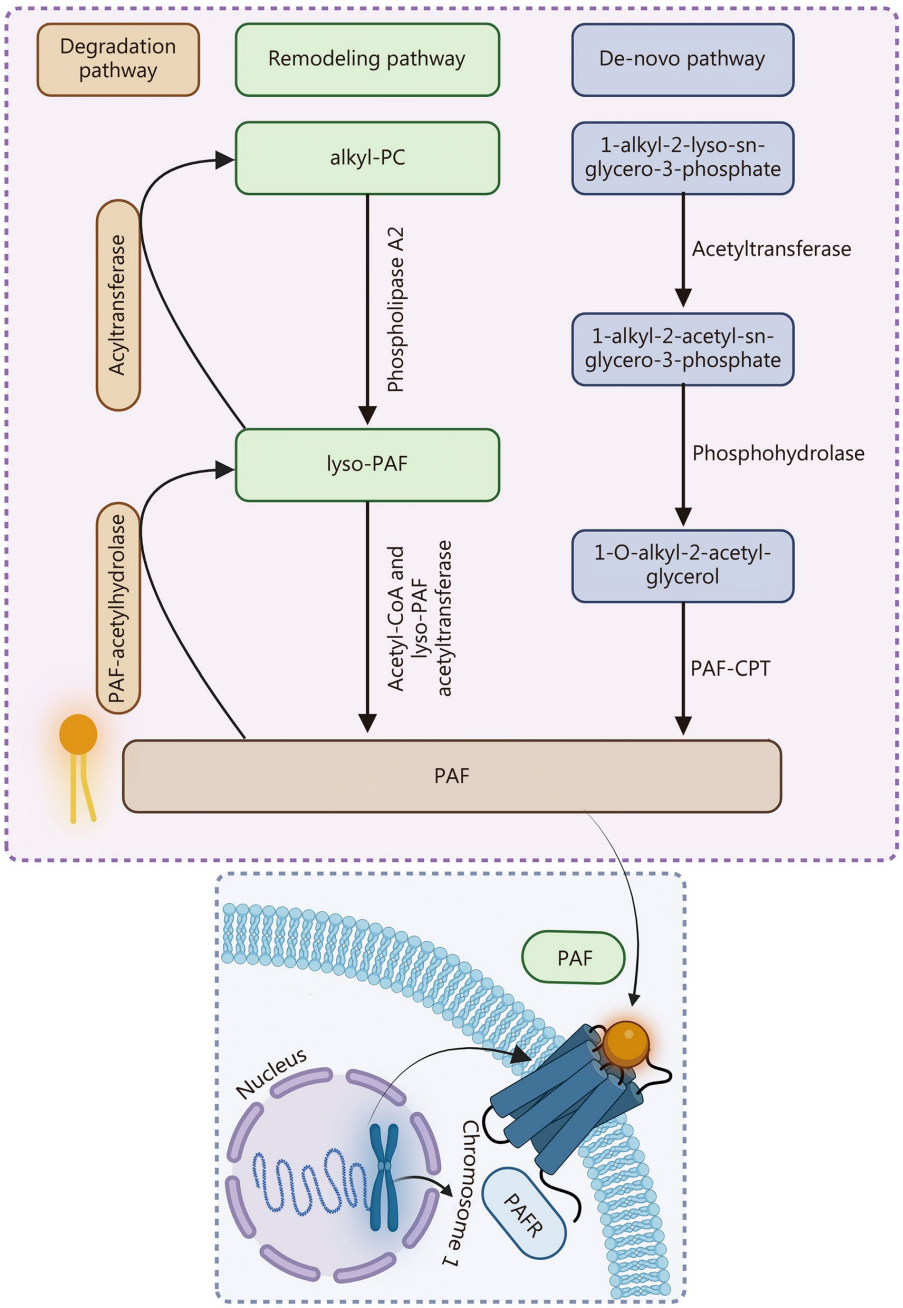
Synthetic pathways and degradation pathway of platelet-activating factor (PAF). The remodeling pathway involves the interconversion between 1-O-alkyl-2-lyso-sn-glycero-3-phosphocholine (alkyl-PC) and 1-alkyl-2-lyso-sn-glycero-3-phosphocholine (lyso-PAF), where alkyl-PC is deacylated by phospholipase A2 to form lyso-PAF, and lyso-PAF is re-acetylated by an acetyltransferase to form PAF. The de-novo pathway begins with the acetylation of 1-alkyl-2-lyso-sn-glycero-3-phosphate by acetyltransferase to form 1-alkyl-2-acetyl-sn-glycero-3-phosphate. This intermediate is then converted to 1-O-alkyl-2-acetyl-glycerol by phosphohydrolase, and finally, PAF is synthesized through the action of PAF-choline phosphotransferase (PAF-CPT). In the degradation pathway, PAF is hydrolyzed by PAF-acetylhydrolase to produce lyso-PAF, which can then be converted to alkyl-PC by acyltransferase. PAFR platelet activating factor receptor

Known chemically as acetyl-glyceryl-ether-phosphorylcholine, PAF is an ether phospholipid with remarkable potential as a lipid chemical mediator [26, 27]. Notably, PAF belongs to a family of naturally occurring pro-inflammatory lipids that have been implicated in the development of cancer and other inflammatory diseases, including cardiovascular disease, allergic reactions, elevated leukocyte adhesion, chemotaxis, degranulation, respiratory burst, and increased vascular permeability [28–31]. Importantly, as an intercellular messenger, PAF exerts its broad pathophysiological effects at concentrations as low as 10^–12^ M [32, 33]. Initially, the term PAF was intended to describe a specific phosphoglycerylether lipid, 1-O-alkyl-2-acetyl-sn-glycero-3-phosphocholine, which features an ether-linked fatty chain consisting of 16 to 18 carbon atoms at the sn-1 position of the glycerol backbone [26], as illustrated in Fig. 1.

The ether bond in the sn-1 position of PAF has been preserved because of its significant biological role, in contrast to the majority of ether lipids, which have evolved to replace their ether bond with esterified equivalents [34]. Furthermore, the term PAF encompasses a group of molecules exhibiting PAF-like biological functions, categorized into two main groups. The first group includes phospholipid molecules structurally similar to classical PAF, such as 1-O-alkyl/acyl/alkenyl-2-acetyl/acyl-sn-glycero-3-phosphocholine, referred to as PAF-like lipids [35, 36]. The second group comprises molecules that demonstrate comparable biological activities to PAF but possess partially similar or dissimilar structures relative to the classical PAF, designated as PAF-like activity molecules [37].

PAF is synthesized by various cell types, including platelets, neutrophils, monocytes/ macrophages, basophils, eosinophils, mast cells, and endothelial cells. Additional evidence indicates its production by structural cells such as cardiomyocytes, vascular endothelial cells, urothelial cells, airway epithelial cells, and human gastric and endometrial adenocarcinoma cell lines [11, 36, 38–40]. The functions of PAF encompass both physiological and pathological aspects, primarily determined by the level of PAF production and enzymatic regulation [16, 38].

The synthesis of PAF is achieved through two distinct enzymatic mechanisms, namely the remodeling and de novo pathways [41, 42]. The initial biosynthetic pathway involves the process of remodeling, wherein phospholipase A2 (PLA2) converts ether analogs of phosphatidylcholine into an inactive 1-alkyl-2-lyso-sn-glycero-3-phosphocholine (lyso-PAF) [43, 44]. This lyso-PAF is subsequently acetylated to form active PAF by isoforms of acetyl-CoA and lyso-PAF acetyltransferases (Lyso-PAF ATs, EC 2.3.1.67), particularly lysophosphatidylcholine acyltransferase (LPCAT) 1 and LPCAT2 [43, 44]. According to the most recent revision and nomenclature proposal for mammalian lysophospholipid acyltransferases (LPLATs), LPLAT8 (LPCAT1), also known as (AGPAT9), AGPAT10, or acyltransferase-like 2 (AYTL2), is expressed in the lung, retina, and various other organs, where it synthesizes C16:0-containing phosphatidylcholine (PC) [45–47]. Notably, C2:0 (acetic acid) is incorporated into lyso-PAF by LPLAT9 (LPCAT2), also referred to as AGPAT11 or AYTL1, to generate PAF [43, 48, 49].

The de novo pathway represents the second major mechanism for PAF biosynthesis, which commences with the acetylation of 1-alkyl-2-lyso-sn-glycero-3-phosphate by acetyltransferase [50]. Subsequently, a phosphohydrolase acts sequentially, followed by the specific activity of dithiothreitol-insensitive cytidine 5’ diphosphocholine (CDP-choline): PAF choline phosphotransferase (PAF-CPT, EC 2.7.8.2), which incorporates CDP-choline into 1-O-alkyl-2-acetyl-glycerol, resulting in the formation of PAF [50]. In contrast to the remodeling pathway, which produces PAF in response to inflammatory triggers and is primarily implicated in inflammatory cascades, it is theorized that the de novo pathway acts as the mechanism for endogenous PAF generation to maintain physiological concentrations [51, 52]. Once synthesized, PAF is degraded by the degradation pathway involving PAF-acetylhydrolase enzymes, which metabolize active PAF to inactive lyso-PAF, which can then be converted to acyl-PAF by LPCAT. The schematic representation of the synthesis and degradation pathways of PAF is shown in Fig. 1.

Importantly, PAF biosynthesis, triggered by extracellular signals, has been documented in various cell types. For instance, it has been observed in mouse peritoneal cells stimulated by calcium ionophore [53], rat peritoneal cells induced by PAF [54], human eosinophils activated by formyl-methionyl-leucyl-phenylalanin (fMLP), a synthetic tripeptide mimicking soluble bacterial factors [55], human mesangial cells activated by lipopolysaccharides (LPS) [56], and IC-21 mouse peritoneal macrophage cells activated by LPS [57, 58]. Notably, Wykle et al. [59] were the first to partially describe the lyso-PAF acetyltransferase enzyme. While the exact mechanisms of this enzyme’s activation remain poorly understood, it has been observed that phosphorylation modulates its activity in human neutrophils [60] and rat splenic microsomes [58, 61, 62]. A study conducted in 2005 examined the priming effect of LPS on PAF-induced acetyltransferase activation in toll-like receptor 4 (TLR4)-knockout (KO), myeloid differentiation primary response protein 88 (MyD88)-KO, and Toll/IL-1R domain-containing adaptor inducing IFN-β (TRIF)-KO mice [58]. This report demonstrated that lyso-PAF acetyltransferase was activated by the following mechanisms: 1) a second-order time course following PAFR stimulation; 2) a minute-order time course following LPS stimulation in a MyD88- and p38 mitogen-activated protein kinase (MAPK)-dependent manner; and 3) an hour-order time course following LPS stimulation in a MyD88- and TRIF-independent manner [58].

Of note, it was discovered in 2007 that the lyso-PAF acetyltransferase enzyme is responsible for producing membrane glycerophospholipids, which serve as important membrane components and precursors to PAF [43]. Under resting conditions, this enzyme synthesizes membrane lipids using arachidonoyl-CoA [43]. However, its acetyltransferase activity was found to be enhanced when PAF synthesis increased in response to acute inflammation induced by TLR4 activation [43]. Moreover, it has been demonstrated that rapid PAF production in response to a non-hydrolyzed analog of PAF (methylcarbamyl PAF) or ATP stimulation necessitates protein kinase C alph-dependent Ser-34 phosphorylation of LPCAT2 [63]. In contrast, phosphorylation of the same residue after 30 min of LPS stimulation is facilitated by the p38 MAPK/MAPK-activated protein kinase 2-dependent pathway [64].

PAF plays an essential role in many physiological activities, including the mediation of normal inflammatory responses, regulation of blood circulation and pressure, modulation of coagulation responses, glycogen degradation, brain function, reproduction, fetal implantation, lung maturation, initiation of parturition, wound healing, apoptosis, angiogenesis, and exocrine gland functions [16, 65–70]. PAF can be considered a dualistic entity, as it is believed to have originated as a component of the innate immune system’s protective mechanism, yet it also contributes to the development of uncontrolled inflammatory pathological conditions [71]. Excessive levels of PAF have been linked to the development of various chronic disorders characterized by inflammation, leading to conditions such as allergy, asthma, diabetes, renal diseases, cancer, Chagas disease, stroke, sepsis, human immunodeficiency viruspathogenesis, severe acute respiratory syndrome coronavirus 2 infection, periodontitis, chronic rhinosinusitis with nasal polyps, and neuropathic pain [29, 31, 33, 48, 51, 72–84].

**Fig. 2:**
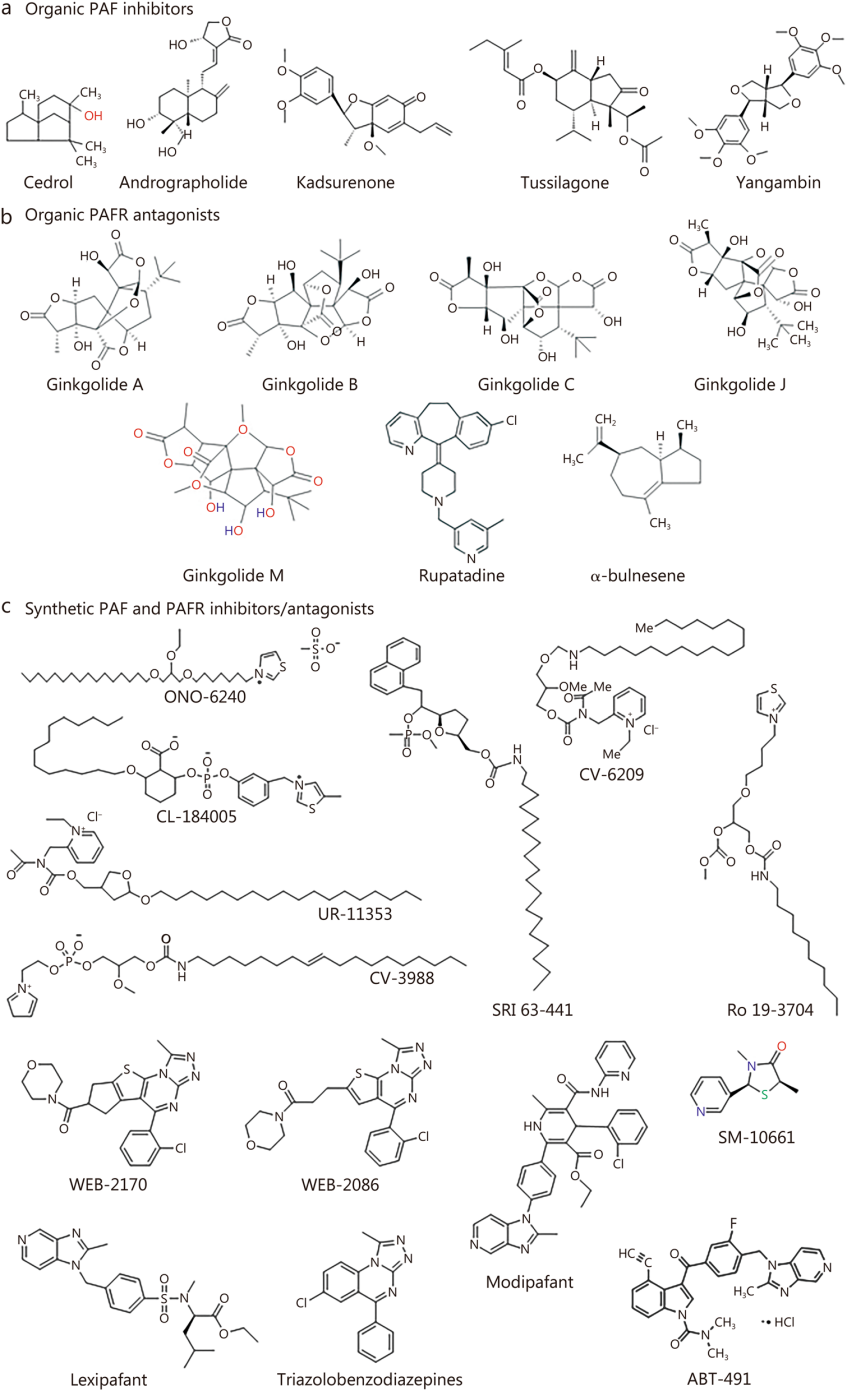
The chemical structures of organic and synthetic platelet-activating factor (PAF) and platelet activating factor receptor (PAFR) inhibitors/antagonists. **a** Organic PAF inhibitors include cedrol, andrographolide, kadusuerone, tussilagone, and yangambin. **b** Organic PAFR inhibitors features ginkgolide A, ginkgolide B, ginkgolide C, ginkgolide J, ginkgolide M, rupatadine, and α-bulnesene. **c** Synthetic PAF and PAFR inhibitors/antagonists depict ONO-6240, CL-184005, UR-11353, CV-3988, SRI 63–441, CV-6209, Ro 19–3704, WEB-2170, WEB-2086, lexipafant, triazolobenzodiazepines, modipafant), SM-10661, and ABT-491

The majority of PAF-mediated effects can be attributed to structure–activity relationships, which indicate that the chiral center and O-acetyl group at the C_2_ position are crucial for biological activity. Replacing the acetyl group with propyl, isobutyl or longer side chains greatly reduces its biological activity. Additionally, the phosphate group of the polar head region of PAF, which is attached to the quaternary ammonium group via an ethyl bridge, is highly sensitive to alterations. Substituting this quaternary ammonium group with an uncharged amino group or replacing its methyl groups with ethyl, propyl, allyl, or carboxymethyl groups also diminishes PAF’s biological activity [84]. Furthermore, the following modifications reduce the biological activity of PAF: 1) substitution of the phosphate group by uncharged butoxy or sulfonylbismethylene groups; 2) elongation of the ethyl bridge; 3) elimination of the oxygen atom linked to the phosphate group; and 4) substitutions of multiple oxygen atoms in the alkoxy chain. Overall, these findings suggest that PAF analogs possessing 1-alkyl/1-acyl side chains (without the aforementioned modifications) can bind to and activate PAFRs in various cell types. The biological activity of PAF can be regulated or blocked by various PAF and PAFR inhibitors/antagonists, as shown in Fig. 2.

## PAFR

The biological functions of PAF are mediated by G-protein-coupled receptors (GPCRs) known as PAFRs, which are expressed on the plasma membrane of numerous mammalian cells, including endothelial cells, neutrophils, monocytes, dendritic cells, platelets, and leukocytes [16, 27, 85]. The initial cloning of a receptor for a lipid mediator revealed that the PAFR belongs to the GPCR superfamily, offering insight into the intricate signal transduction mechanisms activated by PAFR stimulation [86]. Notably, PAFRs are also expressed in many tissues, including the lungs, spleen, heart, kidneys, skeletal muscle, and blood cells [87].

The human PAFR is localized on chromosome 1 [88]. Upon interaction with PAF and PAF-like molecules, the PAFR activates numerous intracellular signaling pathways, resulting in autocrine, endocrine, paracrine, and juxtacrine cellular activities [16]. Additionally, PAFR coupling with the Gq protein initiates downstream signaling cascades [89]. A wide variety of compounds have been classified as inverse agonists and antagonists of the PAFR, exhibiting diverse effects on receptor structure and function [90]. The PAFR exhibits a conventional GPCR architecture, comprising a helical bundle of seven transmembrane helices (I-VII). Conformational changes in this helical bundle, which are ligand-dependent, play a pivotal role in PAFR activation [91]. Notably, while the precise configuration of the PAF binding site on the PAFR is not yet known, molecular docking study using the corresponding three-dimensional structure has confirmed the formation of the PAF/PAFR complex (91). Improper activation of the PAFR pathway has been associated with inflammation and inflammatory pathological conditions [16, 92].

## PAF and PAFR in cancer

PAF/PAFR signaling plays a pivotal role in oncogenic transformation, thrombosis, carcinogenesis, anti-apoptosis, metastasis, angiogenesis, and the progression of various types of cancers [51, 75, 93–95]. The presence of PAF in the tumor microenvironment may be attributed to the activated endothelial cells and/or malignant cells themselves, as evidenced by multiple studies that have highlighted the capacity of different cancer cell types to generate PAF and express PAFR on their cell membranes [96–110]. Hence, given the intrinsic nature of cancerous cells to produce PAF and exhibit PAFR expression, PAF exerts a crucial influence on cancer development [94]. Additionally, many cytokines and growth factors, including vascular endothelial growth factor (VEGF), fibroblast growth factor (FGF), and tumor necrosis factor-α (TNF-α), have been found to promote the synthesis of PAF in cancer cells [51].

Notably, recent investigations have established a significant association between PAF synthesis and PAFR expression and the aggressiveness of cancer cells [51, 109, 111]. By changing the local cytokine and angiogenic networks, PAF also contributes significantly to immune system suppression, metastasis, and tumor growth [96, 112, 113]. Upon its release into the tumor microenvironment, PAF has the potential to exert an impact on the endothelial cells through either an autocrine or a paracrine mechanism. Elevated levels of circulating PAF elicit a rapid inflammatory reaction characterized by augmented endothelial permeability and other notable biological effects [51]. These include 1) heightened expression of PAFR and PAF production by endothelial cells, platelets, and cancer cells; 2) stimulation of cellular proliferation; 3) activation of cyclooxygenase type 2 enzyme (COX-2), resulting in prostaglandin synthesis; and 4) induction of metalloproteases and serine proteases via Janus kinase (JAK) and signal transducers and activators of transcription (STATs) signaling pathways, ultimately leading to extracellular matrix degradation.

Here, we discussed the implication of PAF’s involvement in various malignancies. The roles and mechanisms of PAF/PAFR signaling in various cancer types are summarized in Table 1 [110, 114–126].

**Table 1:** Summary of the roles and mechanisms of PAF/PAFR signaling in various cancer types

Cancer type	Role(s) of PAF/PAFR signaling	Mechanism(s)	References
Breast cancer (BC)	Promoting bone metastases	Inhibiting NF-κB activation and osteoclast differentiation via blocking NFATc1 transcription activity	[114]
	Inducing BMMs differentiation		
	Initiation of transformation in non-tumorigenic breast epithelial cells	Disrupting the overall shape of the spheroids	[115]
	Facilitation the migration of metastatic BC cells	Mediated by the PI3-kinase and/or JNK pathways	
Ovarian cancer (OC)	Facilitation the formation of spheroids	PAF/PAFR signaling pathway	[116]
	Prevention of the transition of dormant OC cells		
	Increased expression of stemness genes		
	Promotion of the proliferation of non-mucinous OC in vitro and in vivo by MSCs	PAF/PAFR signaling pathway	[117]
	Promotion of the progression of OC	Production of PAF induced by EGF leads to a positive feedback loop, whereby the activation of the PAF/PAFR signaling pathway	[118]
	Reduced overall survival as well as a shorter duration of recurrence-free survival	High cytoplasmic PAFR expression	[119]
Lung cancer	Enhancing the proliferation of non-small cell lung cancer (NSCLC) cell lines	Activation of functional PAFR	[120]
	Enhancing the growth and spontaneous metastasis of lung tumors	Systemic activation of PAFR	[121]
	Promoting invasion and metastasis of human NSCLC cells	Positive reciprocal relationship between the PAFR and STAT3 signaling pathways	[122]
Skin cancer	Mediating immune suppression	Migration of mast cells from the skin to the lymph nodes (LNs)	[123]
	Skin cancer induction	Disruption of DNA repair	[124]
Oral cancer	Stimulation of tumoral development, invasiveness, and migration in cell lines derived from oral squamous cell carcinoma (OSCC)	Possible interaction between LPCAT1 and the PAF/PAFR pathway	[125]
Prostate cancer	Promoting the growth, invasion, and metastasis of prostate cancer cells	Activation of the ERK1/2 pathway results in increased MMP-3 expression and decreased E-cadherin expression	[110]
Esophageal Squamous cell Carcinoma	Modification of tumor microenvironment	PAFR/STAT3 axis facilitates tumor growth	[126]

*PAF* platelet-activating factor, *PAFR* platelet-activating factor-receptor, *NF-κB* nuclear factor kappa-B, *BMMs* bone marrow monocytes, *NFATc1* nuclear factor of activated T-cells, cytoplasmic 1, *PI3K* phosphoinositide 3-kinase, *JNK* c-Jun N-terminal kinase, *MSCs* mesenchymal stem cells, *EGF* epidermal growth factor, *LPCAT1* lysophosphatidylcholine acyltransferase 1, *ERK* extracellular signal-regulated kinase, *MMP* matrix metalloproteinase, *STAT3 s*ignal transducer and activator of transcription 3

### Breast cancer (BC)

BC is the most prevalent malignant tumor in women [127–129]. Metastasis significantly contributes to the elevated mortality rates observed in BC patients [130]. While bones, liver, lungs, and brain are common sites of metastasis in BC, bones are the most frequently affected organs, whereas the brain is the least commonly affected [130–134].

In 2018, a study revealed a correlation between the upregulation of PAFR and an increased incidence of bone metastases in BC [114]. Additionally, it was demonstrated that PAF significantly contributes to enhanced BC cell migration and BC-induced osteoclastogenesis [114]. Furthermore, PAF plays a crucial role in promoting bone metastases in BC and inducing the differentiation of bone marrow monocytes (BMMs) through inhibiting nuclear factor kappa-B (NF-κB) activation, and the differentiation of osteoclast via blocking nuclear factor of activated T-cells, cytoplasmic 1 (NFATc1 transcription activity [114].

In a study, Anandi et al. [115] provided evidence for the potential involvement of PAF in the initiation of transformation in non-tumorigenic breast epithelial cells cultivated as spheroids. PAF was shown to disrupt the overall shape of the spheroids and induce proliferation, a hallmark of transformation. Additionally, PAF is crucial for promoting the migration of BC cells, potentially mediated through the c-Jun N-terminal kinase (JNK) and/or phosphoinositide 3-kinase (PI3K) pathways [115].

### Ovarian cancer (OC)

Cancer stem cells (CSCs) represent a distinct subset of tumor cells characterized by their self-renewal ability, clonal tumor initiation, and potential for long-term repopulation [135, 136]. Growing evidence indicates that CSCs play a significant role in tumor recurrence, metastasis, and chemoresistance [137–139]. According to a study conducted by Gao et al. [116], PAF has the potential to facilitate the formation of spheroids and impede the transition of dormant OC cells into the cell cycle. Additionally, there was a notable increase in the proportion of CSCs and upregulate the expression of genes associated with stemness in the group treated with PAF [116]. Consequently, it has been observed that the stemness of osteosarcoma cells can be modulated by PAF via the PAF/PAFR pathway [116].

Furthermore, numerous proteins that maintain stemness have been shown to be elevated to varying degrees. These include retinol-binding protein 4 (RBP4)-stimulated by retinoic acid 6 (STRA6) [140], insulin-like growth factor-1 receptor (IGF-1R) [141], bone morphogenetic protein (BMP) 4 [142], BMP7 [143], v-erb-b2 avian erythroblastic leukemia viral oncogene homolog 2 (ErbB2) [144], ErbB3 [145], IGF binding proteins 1 and 2, and angiopoietin-like protein 3 (ANGPTL3) [146], vascular endothelial growth factor receptor-2 (VEGFR2)/signal transducer and activator of transcription 3 (STAT3) [147], uPAR [148], disintegrin and metalloproteinase domain-containing protein 12 (ADAM12) [149], receptor activator of nuclear factor kβ (RANK)/RANK ligand (RANKL)/osteoprotegerin (OPG) [150], and matrix metalloproteinase (MMP)-10 [151]. Multiple studies have indicated that the primary canonical pathway associated with these proteins involves the BMP and ErbB receptor families [116]. Specifically, BMP signaling plays a crucial role in the maintenance and development of CSCs by affecting their functional characteristics such as self-renewal, chemoresistance, and tumor-initiating abilities across various cancer models, including OC. Notably, the ErbB family, particularly the epidermal growth factor receptor (EGFR), significantly contributes to cancer stemness via interactions with IGF-1R, activation of MAPKs, and phosphorylation of Akt in many malignancies [116]. Gene Ontology (GO) analysis and Kyoto Encyclopedia of Genes and Genomes (KEGG) pathway enrichment analysis revealed that the majority of proteins supporting CSC functions are concentrated in the MAPK and the PI3K/protein kinase B (Akt) signaling pathways [116].

Notably, the same group demonstrated that mesenchymal stem cells (MSCs) facilitated the proliferation of OC cells both in vitro and in vivo, with this effect being attributed, at least in part, to the activation of the PAF/PAFR signaling pathway [117]. The study revealed that MSCs within the tumor microenvironment exhibited a notable secretion of PAF [117]. Specifically, the authors [117] showed that MSCs present within the tumor microenvironment released elevated levels of PAF, and MSC-enriched conditioned medium (CM) promoted the proliferation and migration of non-mucinous OCCs. Importantly, treatment with PAF and MSC-CM resulted in markedly elevated activation and expression of the focal adhesion kinase (FAK) and cyclin D1 in OCCs [142]. These findings align with previous research indicating that various downstream targets of PAF/PAFR signaling, such as FAK, PI3K, and MMP2, are associated with OCC proliferation and invasion [152].

Along similar lines, the stimulation of PAF release from human OC cells was found to be facilitated by epidermal growth factor (EGF) through its interaction with the EGFR and subsequent transactivation of the PAFR [118]. It is suggested that the production of PAF induced by EGF may lead to a positive feedback loop, whereby the activation of PAFR further promotes OC progression [118]. Moreover, another study examined the long-term impact of PAFR expression on OC patient outcomes. The findings revealed a significant correlation between high cytoplasmic PAFR expression and poorer overall survival, as well as a shorter duration of recurrence-free survival [119].

### Lung cancer

In a recent study, the significance of PAFR expression in A549 and H1299 non-small cell lung cancer (NSCLC) cell lines was re-evaluated [120]. The researchers [120] utilized a well-established PAFR agonist, carbamyl-PAF (CPAF), to explore its effects on cell proliferation in vitro. The results demonstrated that CPAF treatment significantly increased the proliferation of both A549 and H1299 cell lines in a dose-dependent manner [120]. Therefore, the activation of functional PAFR enhances the proliferative capacity of NSCLC cell lines [120]. Consistent with these findings, a study conducted by Hackler et al. [121] disclosed that the systemic activation of PAFR promotes the growth and spontaneous metastasis of lung tumors, mediated by the activation of the host PAFR [121].

In a study, Chen et al. [122] demonstrated a correlation between the upregulation of PAFR expression and the progression and unfavorable prognosis of NSCLC. The researchers employed immunohistochemistry (IHC) techniques to assess PAFR expression in 150 NSCLC tumor samples. Their analysis revealed a substantial increase in PAFR protein levels in 56% of the NSCLC samples (84 out of 150) compared to adjacent normal lung tissues. Furthermore, the study found that NSCLC patients with high PAFR expression exhibited shorter overall survival rates, and PAFR expression was positively associated with distant metastasis in NSCLC patients. This study presents a significant discovery that PAFR plays a crucial role in promoting invasion and metastasis of human NSCLC cells [122], as observed in both in vitro and in vivo experimental models. The mechanism underlying this promotion involves the regulation of epithelial-mesenchymal transition (EMT) [122]. Specifically, the activation of the PAF/PAFR signaling pathway stimulated the STAT3 pathway, which is primarily responsible for regulating tumor growth [122]. Additionally, a positive reciprocal relationship between the PAFR and SATA3 was identified, further contributing to the promotion of invasion and metastasis of NSCLC [122]. Consistent with these findings, the activation of host-PAFR signaling has been shown to hinder the effectiveness of cancer treatments in multiple experimental tumor models [23, 153, 154].

### Skin cancer

The induction of skin cancer is a significant concern associated with immunosuppression caused by ultraviolet (UV) radiation [155, 156]. Numerous studies have documented that PAF plays a pivotal role in UV-induced skin cancer [157–159]. Keratinocytes exposed to low doses of UV radiation exhibit increased PAF production [160, 161]. This release of PAF then leads to the migration of mast cells from the skin to the lymph nodes (LNs), where they contribute to immune suppression [124]. Notably, PAF serves two distinct roles. First, it supports apoptosis and maintains homeostasis in healthy skin [123]. Second, PAF functions as a mediator of inflammation and can block DNA repair, leading to the formation of skin cancer in persistently irradiated, inflamed skin where UV-induced inactivation of normal tumor suppressor pathways (i.e., p53, phosphatase, and tensin homolog) occurs [123].

### Other cancers

In oral cancer, a study has demonstrated the significance of the PAF/PAFR pathway in association with the overexpression of the LPCAT1 enzyme, a membrane surface protein involved in the remodeling of phosphatidylcholine metabolism [125]. This overexpression has been shown to contribute to the stimulation of tumorigenesis, invasiveness, and migration in cell lines derived from oral squamous cell carcinoma (OSCC) [125].

In prostate cancer, Ji et al. [110] discovered that PAFR activates the extracellular signal-regulated kinases (ERK) 1/2 pathway, leading to an increase in MMP-3 and a decrease in E-cadherin expression. These changes promote the growth, invasion, and metastasis of prostate cancer cells, indicating that PAFR may serve as a potential therapeutic target for the treatment of prostate cancer [110].

In esophageal squamous cell carcinoma, Zhao et al. [126] proposed that the modification of the tumor microenvironment, particularly through cancer-associated fibroblasts (CAFs), facilitates the growth of malignant tumors via PAFR/STAT3 signaling. Specially, two cytokines, interleukin (IL)-6 and IL-11, which are related to STAT3 signaling, mediate the communication between CAFs and tumor cells along the PAFR/STAT3 axis. This process can be inhibited pharmacologically by targeting PAFR and STAT3 [126].

## PAF and PAFR inhibitors

Several PAF and PAFR inhibitors have been characterized and can be categorized based on three main criteria: 1) their origin, which can be either natural or chemically synthesized; 2) their chemical structure, encompassing nitrogen heterocyclic compounds, PAF analogs (molecules structurally similar to PAF), dihydropyridines, natural medicines, and other compounds; or 3) their mode of interaction with the PAFR, distinguishing between specific and nonspecific inhibitors [81]. It is important to note that the terms “inhibitors” or “antagonists” are often used interchangeably in the literature; however, they refer to agents with specific activities towards PAF or PAFR. Herein, we categorize these inhibitors according to their sources and provide selected examples.

### Organic PAF inhibitors

Cedrol, a type of naturally occurring sesquiterpene alcohol, is widely distributed throughout the plant kingdom and is particularly abundant in conifers such as *Cedrus atlantica* and *Juniperus virginiana* [162, 163]. Cedrol is recognized for its diverse pharmacological properties, including antioxidant and analgesic [164], anti-inflammatory [165], antibacterial [166], sedative [167], hair growth-promoting [168], PAF antagonist [169], and antitumor effects [170].

Kadsurenone is a naturally occurring compound extracted from the stems of *Piper kadsura*. It is widely utilized in traditional Chinese medicine for the treatment of conditions such as asthma and rheumatoid arthritis (RA) [171]. Kadsurenone has been shown to possess potent inhibitory effects against PAF, thereby effectively mitigating PAF-induced adverse reactions [172].

Tussilagone, another naturally occurring compound derived from *Tussilago farfara*, has been traditionally used in oriental medicine as a folk remedy for managing pulmonary inflammatory diseases. Several studies have reported its anti-inflammatory properties in various human organ systems experiencing inflammation [173–176]. Tussilagone functions as a nonselective inhibitor of PAF by blocking calcium channels [177].

*Ocotea duckei Vattimo*, commonly known as “louro-de-cheiro”, is a botanical species belonging to the Lauraceae family and is primarily distributed in the northeastern region of Brazil. Furofuran lignan, including yangambin, and other lignoids have been isolated from its leaves [178, 179]. Yangambin exhibits PAF antagonistic activity, demonstrating anti-allergic and analgesic properties [180]. Research findings indicate that this PAF inhibitor acts as a competitive antagonist by inhibiting the binding of PAF to its receptor, PAFR [181].

### Organic PAFR antagonists

*Ginkgo biloba*, a member of the Ginkgoaceae family, is a widely recognized ancient tree that has been utilized as a medicinal herb in both traditional Chinese and Western medicine for centuries [182]. In recent years, researchers have successfully isolated and purified various diterpene lactones, including ginkgolides A, B, C, J, and M, as well as flavonoids from *Ginkgo biloba* [183]. These terpene lactones have been reported to possess anti-inflammatory [184, 185], endothelial protective [186, 187], cardioprotective [188], anti-platelet [189], and antioxidant properties, as demonstrated in both cell-based and animal-based studies [190, 191]. Notably, ginkgolides A, B, and C also function as PAFR antagonists [192–197].

Ginkgolide B demonstrates a potent inhibitory effect on inflammation and platelet activation by suppressing elevated PAF levels and diminishing the interaction between PAF with PAFR [198, 199]. Furthermore, it has been documented that PAF facilitates platelet activation [200], whereas GB pretreatment effectively counteracts this effect [201–203]. Notably, Vogensen et al. [204] synthesized various ginkgolide B derivatives with alterations at the 7-position and conducted pharmacological evaluations of these compounds using cloned PAFRs, validating their efficacy as PAFR antagonists. Moreover, Strømgaard et al. [205] analyzed the effects of terpene trilactones (TTLs) isolated from *Ginkgo biloba* extracts on the cloned PAFR. Their data revealed that among the native compounds, ginkgolides A and B exhibited the highest potency as PAFR antagonists. Besides, several other analogs of ginkgolide were synthesized, and PAFR binding experiments indicated that the majority of these analogs displayed greater antagonistic activity compared to their parental compounds, presenting intriguing possibilities for the forthcoming investigations into ginkgolides’ interactions with PAFR [205].

Importantly, in addition to blocking PAF and histaminic receptors, the second-generation antihistamine medication rupatadine has been shown to possess PAFR antagonistic activity, as well as anti-inflammatory, anti-allergic, and anti-fibrotic properties [206, 207]. Furthermore, the essential oil of Pogostemon cablin contains a small oily sesquiterpene compound called α-bulnesene, which has been isolated and has demonstrated a highly potent inhibitory effect on platelet aggregation [208]. In 2006, Hsu et al. [208] evaluated the anti-platelet mechanism of α-bulnesene, confirming its efficacy as a PAFR antagonist.

### Synthetic PAF and PAFR inhibitors/antagonists

In this category, CV-3988, a thiazolium derivative identified as a zwitterionic compound, served as the pioneering synthetic antagonist of PAF [209]. Additionally, CV-3988 [209, 210] and CV-6209 [211], ONO-6240 [212], and Ro 19-3704 [213] represent the initial compounds that were synthesized and exhibited structural resemblance to PAF. Subsequent advancements in the field involved the substitution of the glycerol backbone with cyclic structures, exemplified by inhibitors such as SRI 63-441 [214], SRI 63-073 [215], UR-11353 [216], and CL-184005 [217].

Subsequently, the synthesized PAF inhibitors exhibited distinct structural characteristics in comparison to PAF. These antagonists consist of heterocyclic structures characterized by the presence of an sp2 nitrogen atom, which acts as a hydrogen bond acceptor and interacts with the PAFR [81]. Notable examples include pyrrolothiazole-related antagonists such as tulopafant [218], thiazolidine derivatives like SM-10661 [219], imidazolyl derivatives such as modipafant [220] and lexipafant [221], and hetrazepine derivatives, including WEB-2086 and WEB-2170 [222]. Moreover, numerous synthetic PAF and PAFR inhibitors/antagonists have been developed, such as psychotropic triazolobenzodiazepines [223], L-652731 [224], and a range of inorganic metal complexes [225, 226]. It is to be noted that WEB-2086 has been used as a specific PAFR antagonist [120] and has been shown to ameliorate neuropathic pain in mice [83]. Notably, Y-24180, the modipafant (UK-80067)—( +)-enantiomer of UK-74505, UK-74,505, and SR27417A are additional examples of PAF inhibitors and PAFR antagonists [73]. Moreover, ABT-491 is a potent PAFR antagonist that effectively inhibits PAFR-mediated responses at nanomolar and sub-nanomolar concentrations in platelets and neutrophils, which are critical to PAF pathophysiology [227].

Synthetic alkyl phospholipids, which are structurally similar to PAF, represent a novel category of anticancer medicines [228, 229]. These molecules are synthetic analogs of natural phosphatidylcholines, in which ester linkages are replaced by increased metabolically stable ether bonds, thereby enhancing their therapeutic potential [84]. It has been shown that ether phospholipids may permeabilize and fluidize tumor cell membranes [230, 231]. One such compound is methoxy-substituted 1-O-octadecyl-2-O-methyl-rac-glycero-3-phosphocholine (ETI8-OCH3 or Edelfosine) [232]. PAF consists of four parts: a glycerol backbone with 2-O-acetyl, 1-O-alkyl ether, and 3-phosphocholine side chains [233]. The replacement of 2-O-acetyl group in PAF with a 2-O-methyl group in edelfosine results in an immunomodulatory molecule that suppresses tumor cell growth [84, 234]. A notable feature of alkyl phospholipids is their capacity to induce apoptosis in rapidly proliferating tumor cells, with edelfosine being recognized as a highly efficient prototype in many anticancer activity studies [233, 235, 236]. Edelfosine, a non-mutagenic molecule that selectively targets malignant cells without harming normal ones, does not interfere with the S and M phases of the cell cycle. It can be administered orally and serves as a promising prototype for anticancer drug development [237–241]. In 1993, Diomede et al. [232] demonstrated that edelfosine neither binds to the PAFR nor inhibits PAF from attaching to its receptor in HL60 human promyelocytic leukemia cells. Despite structural similarities to PAF, ether phospholipids do not interact with the PAFR on rabbit platelet membranes [232].

Regarding the mechanisms of action of edelfosine, several investigations indicate that it may induce the activation of Fas death receptors independently of a conventional receptor-mediated pathway initiated by ligand-receptor contact [242]. Instead, this activation involves the translocation and co-clustering of Fas receptors into membrane rafts mediated by alkyl phospholipids, suggesting that these receptors can be activated without a traditional ligand-receptor interaction [242]. Nonetheless, another hypothesis posits that following cellular uptake facilitated by membrane rafts [17], alkyl phospholipids inhibit the cytidine triphosphate (CTP): phosphocholine cytidyltransferase (CCT) enzyme, thereby markedly reducing the biosynthesis of phosphatidylcholine and eventually inducing apoptosis in cancerous tumor cells [243–250]. Despite substantial evidence supporting CCT inhibition by alkyl phospholipids, no research has yet elucidated the structural basis of this phenomenon. Neto et al. [233] conducted research using molecular docking simulations and assessments of molecular interaction domains to propose the most likely binding mechanisms for four alkyl phospholipids, including edelfosine, to the catalytic domain of human CCT. Their studies suggest potential pathways for developing innovative active alkyl phospholipids, as existing molecules, despite promising preclinical, continue to exhibit limited clinical applicability [242].

Cell cycle progression depends on the availability of phosphatidylcholine, and its depletion can trigger processes leading to cell cycle arrest and apoptosis. The CCT enzyme catalyzes the conversion of choline phosphate to CDP-choline, a precursor essential for the synthesis of phosphatidylcholines [251]. Cell membrane signals that reflect the relative levels of phosphatidylcholine regulate CCT activity [252]. A number of malignancies exhibit increased fatty acid production, which in turn stimulates phospholipid synthesis via the CDP-choline pathway, thereby facilitating rapid tumor cell proliferation [252–256].

Notably, six metabolites were identified when rabbit platelets were treated with alkyl acetyl-G (1-alkyl-2-acetyl-sn-glycerol), one of which is PAF [257]. Importantly, research conducted by Lee et al. [258] demonstrated that saponin-permeabilized rabbit platelets contain a particular dithiothreitol (DTT)-insensitive choline phosphotransferase. DTT increases the synthesis of PAF from alkyl acetyl-G but prevents phosphatidylcholine synthesis from diolein [258]. The authors also showed that the availability of CDP-choline regulates the production of PAF from alkyl acetyl-G [258]. Additionally, the generation of PAF from alkyl acetyl-G is boosted fivefold when rabbit platelets are incubated with sodium oleate, which activates CCT to produce more CDP-choline through its translocation from the cytosol to membranes [258]. Overall, these findings suggest two opposing theories: one positing that edelfosine acts as a PAF agonist, and the other indicating its ability to regulate the growth of cancer cells. Thus, further research using diverse models, including cancer cells, is warranted to precisely delineate the role and mechanisms of edelfosine and establish consistent experimental evidence.

The chemical structures of organic and synthetic PAF and PAFR inhibitors/antagonists are depicted in Fig. 2.

### Inhibitors for PAF synthesis

As previously stated, LPCAT1 and LPCAT2 play crucial roles in PAF biosynthesis. Similar to PAFR antagonists, PAF synthesis inhibitors represent potential candidates for treating PAF-related disorders. Using a high-throughput fluorescence-based assay followed by LC–MS/MS-based secondary analysis, Tarui et al. [259] screened a library of 174,131 compounds to identify highly sensitive LPCAT2-specific inhibitors. Notably, N-phenylmaleimide derivatives emerged as promising LPCAT2 inhibitor candidates, with TSI-01 being one compound that exhibited inhibitory activity against both human and mouse LPCAT2, competitively inhibiting the lyso-PAFAT activity with acetyl-CoA [259]. Importantly, TSI-01 effectively inhibited PAF production in mouse peritoneal macrophages [259].

## Importance of PAF and PAFR inhibitors/antagonists in cancer treatment

Despite the availability of treatment options, including radiation therapy, chemotherapy, targeted therapy, immunotherapy, or a combination of these approaches, achieving complete remission remains obscure [260–262]. This underscores the need to implicate potential target(s) that may enhance the efficacy of therapeutic agents. Although there are currently no cancer treatments that specifically target PAF or PAFR, numerous in vitro and in vivo studies have demonstrated the effectiveness of PAF inhibitors in cancer therapy. The following sections provide evidence supporting the importance of PAF and PAFR antagonists in cancer treatment, and a summary of their mechanisms and efficacy is given in Table 2 [114, 116, 119, 263–291].

**Table 2:** Summary of the mechanisms and efficacy of the PAF and PAFR inhibitors in various cancer models

Cancer type	PAF/PAFR inhibitor(s)	Mechanism(s)	Finding(s)	References
Lung cancer	Ginkgolide C	Attenuation of the STAT3 pathway	Anti-neoplastic effects	[263]
	Cedrol	Cell cycle arrest at the G1 phase and induction of apoptosis	Decrease in cancer cell viability	[264]
		Engaging both the mitochondrial and PI3K/Akt signaling pathways	Suppression of the growth and induction of programmed cell death in lung cancer cells	[265]
	Andrographolide	Modulating autophagy and regulating the expression of PD-L1	Inhibition of the progression of NSCLC	[266]
Colorectal cancer	Ginkgolide C	Inhibition of the Wnt/β-catenin signaling pathway	Inhibition of colon cancer cell proliferation and induction of apoptosis	[267]
	Ginkgolide B	Suppressing angiogenesis	A promising approach for colitis-associated cancer	[268]
	Andrographolide	Inhibiting the Notch pathway, enhancing the production of intracellular ROS, and inducing cell cycle arrest in the G0/G1 phase in SW-480 cells	Impeding the proliferation of colon cancer cells	[269]
		Inhibiting Hedgehog signaling, increasing intracellular ROS production, triggering apoptosis and cell cycle arrest	Repressing the colon cancer cell growth	[270]
		Induction of cell cycle arrest and programmed cell death via augmentation of intracellular ROS	Anticancer potential against colon cancer cells	[271]
		Suppressing the TLR4/MyD88/NF-κB/MMP9 signaling pathway	Inhibit growth and promote apoptosis in human colon cancer cells	[272]
		Exhibiting antagonistic effects on TNF-α and suppressing angiogenesis by inhibiting the NADPH oxidase/ROS/NF-κB and Src/MAPKs/AP-1 signaling pathways	Anticancer therapeutics for colorectal cancer	[273]
Gastric Cancer (GC)	Andrographolide	Inhibition of HIF-1 and PI3K-Akt signaling pathways	Treatment of GC	[274]
		Inhibition of MMP-2/9 activity and upregulation of tissue inhibitors of metalloproteinase (TIMP) as well as apoptosis-associated proteins	Preventing the proliferation, invasion, and metastasis of GC	[275]
		Causes both non-apoptotic and TNF-related apoptosis-inducing ligand (TRAIL)-mediated apoptosis	Antitumor activity	[276]
Ovarian Cancer (OC)	Ginkgolide B and WEB2086	Induction of cell cycle arrest via targeting PAF/PAFR signaling	Inhibition of CSCs and tumor growth	[116]
	Ginkgolide B	Suppression of cell proliferation and the initiation of cell apoptosis	Inhibiting OC cell growth and inducing apoptosis	[277]
	Rupatadine	Impeding the in vitro proliferation and migration of various types of OC cells	Anti-proliferative impact on OC cells	[119]
Breast Cancer (BC)	Andrographolide	Reducing THOC1-promoted cancer stem cell characteristics	Suppressing the malignancy of triple-negative BC	[278]
		Deactivating of the ER-α receptor and the PI3K/Akt/mTOR signaling pathway	Impeding the proliferation of BC cells and triggering cell apoptosis in BC cells	[279]
		Mitochondria-dependent caspase-mediated apoptosis and G2/M cell cycle arrest	Anti-proliferative activity	[280]
		Downregulating the NF-κB signaling pathway and MMP9 expression	Anti-tumor activity	[281]
	Kadsurenone	Inhibition of the PAF/PAFR signaling pathway	Mitigating osteolytic bone metastases resulting from BC	[114]
Bladder Cancer	Ginkgolide B	Blocking ZEB1 protein translation by upregulating miR-223-3p	Decreasing bladder cancer cell invasiveness	[282]
	Andrographolide	Disrupting NF-κB and PI3K/Akt signaling	Reducing bladder cancer cell growth and increasing apoptosis	[283]
Prostate cancer	Andrographolide	Regulation of genes associated with DNA double-strand breaks, including ATM, NBN, BRCA2, BLM, PALB2, and BLM, and encouraging DNA damage	Enhancing anticancer efficacy	[284]
		Targeting the chemokine receptors CXCR3, CXCR7, and cell cycle regulators	Decreasing prostate cancer cell growth	[285]
Glioblastoma	Andrographolide	Regulation of ERK1/2/c-Myc/p53, leading to cell cycle arrest and subsequent activation of the apoptosis signaling pathway	Suppressing of Human brain cancer cell lines (DBTRG-05MG)	[286]
	Cedrol	Enhancing ROS generation and inducing DNA damage response, cell cycle arrest at the G0/G1 phase, and inducting of apoptosis	Inhibiting GBM cell proliferation	[287]
Liver Cancer	GGC	Suppressing the hepatocyte growth factor (HGF)/c-Met signaling pathways	Inhibiting cell proliferation and inducing cell apoptosis	[288]
	Andrographolide derivative ADN-9	Attenuating the VEGF/VEGFR2/ AKT signaling pathway	Reducing tumor growth and metastasis	[289]
Pancreatic Cancer	WEB2086	Host C/EBPδ influences tumor metastasis in a PAFR-dependent manner	Inhibiting B16F10 tumor cell metastasis in WT mice	[290]
			No effect in mice lacking CCAAT/enhancer-binding protein delta (C/EBPδ)	
	Ginkgolide B	Ginkgolide B alone did not exert any effect	–	[291]
	Ginkgolide B with gemcitabine	Ginkgolide B has the potential to improve the sensitivity of pancreatic cancer cell lines to gemcitabine by inhibiting the PAFR/NF-κB pathway	Augmented gemcitabine-mediated suppression of cell proliferation and tumor growth and enhanced cell apoptosis	

*PAF* platelet-activating factor, *STAT3* signal transducer and activator of transcription 3, *PI3K* phosphoinositide 3-kinase*/Akt* protein kinase B, *PD-L1* programmed cell death ligand 1, *NSCLC* non-small cell lung cancer, *ROS* reactive oxygen species, *TL*R4 toll-like receptor 4, *MyD88* myeloid differentiation primary response protein 88, *NF-κB* nuclear factor kappa-B, *MMP* matrix metalloproteinase, *TNF* tumor necrosis factor, *NADPH* nicotinamide adenine dinucleotide phosphate, *Src* proto-oncogene tyrosine-protein kinase, *MAPKs* mitogen-activated protein kinases, *AP-1* activator protein-1, *HIF-1* hypoxia-inducible factor 1, *GC* gastric cancer, *PAFR* platelet-activating factor-receptor, *CSCs* cancer stem cells, *mTOR* mammalian target of rapamycin, *THOC1* THO complex subunit 1, *ATM* Ataxia-Telangiectasia Mutated, *NBN* nibrin, *BRCA2* breast cancer type 2, *BLM* bloom syndrome RecQ-like helicase, *PALB2* partner and localizer of BRCA2, *CXCR* C-X-C chemokine receptor, *ERK* extracellular signal-regulated kinase, *c-Myc* cellular myelocytomatosis oncogene, *VEGF* vascular endothelial growth factor, *VEGFR* vascular endothelial growth factor receptor, *ADN* andrographolide derivative

### Lung cancer

Yang et al. [263] reported that ginkgolide C exerts anti-neoplastic effects by targeting the STAT3 signaling pathway in NSCLC. STAT3 has been shown to facilitate tumor proliferation and viability. Notably, ginkgolide B-induced autophagy in lung cancer cells is dependent on beclin-1, resulting in the suppression of the NLR family pyrin domain-containing 3 (NLRP3) inflammasome [185]. Consequently, ginkgolide B was found to inhibit the growth, invasion, and colony-forming ability of lung cancer cells [185]. In a separate study, Yun et al. [264] provided evidence of the anticancer properties of cedrol. Treatment with cedrol resulted in decreased viability of A549 cells via cell cycle arrest at the G1 phase and increased apoptosis. Importantly, another investigation demonstrated that cedrol effectively hinders the growth of A549 cells and triggers programmed cell death by engaging both the mitochondrial and PI3K/Akt signaling pathways [265]. Similarly, Wang et al. [266] discovered that andrographolide can inhibit the progression of NSCLC by modulating autophagy and regulating the expression of PD-L1.

### Colorectal cancer

Yang et al. [267] demonstrated that ginkgolide C impedes the proliferation of colon cancer cells and promotes apoptosis by inhibiting the Wnt/β-catenin signaling pathway. Additionally, the PAFR antagonist ginkgolide B was found to increase serum PAF-acetylhydrolase (PAF-AH) activity and ameliorate colonic inflammation in mice, leading to a reduction in both the number and size of tumors [268]. Ginkgolide B also induces the expression of VEGF and microvessel density in tumors, suggesting that it may prevent colitis-associated cancer (CAC) by inhibiting angiogenesis [268]. Thus, ginkgolide B represents a promising therapeutic approach for CAC [268].

Similarly, andrographolide has been found to inhibit the proliferation of colon cancer SW-480 cells by suppressing the Notch signaling pathway by downregulating the expression of NOTCH1 and JAGGED [269]. Additionally, andrographolide has been observed to enhance the production of intracellular reactive oxygen species (ROS) and induce cell cycle arrest in the G0/G1 phase in SW-480 cells [269]. Another study demonstrated andrographolide can inhibit the Hedgehog signaling pathway in colon cancer cells, leading to increased intracellular ROS production, apoptosis induction, and cell cycle arrest [270]. Consequently, andrographolide effectively inhibits the development of colon cancer cells [270].

In a separate report, the growth inhibition of colon cancer cells and induction of apoptosis by andrographolide were found through the generation of ROS, resulting in mitochondrial membrane depolarization, caspase activation, nuclear condensation, and DNA fragmentation [271]. In a study, Zhang et al. [272] disclosed that andrographolide can inhibit growth and induce apoptosis in human colon cancer SW620 cells. This effect was achieved by suppressing the TLR4/MyD88/NF-κB/MMP-9 signaling pathway [272]. Moreover, andrographolide exhibits antagonistic effects on TNF-α-induced IL-8 production in HCT116 colorectal cancer cells by inhibiting the nicotinamide adenine dinucleotide phosphate oxidase/ROS/NF-κB and Src/MAPKs/AP-1 signaling pathways [273]. Furthermore, andrographolide effectively suppresses tumor angiogenesis, indicating its promise for the treatment of colorectal cancer [273].

### Gastric cancer (GC)

Similarly, andrographolide exhibits anti-GC activity by targeting multiple signaling pathways and biological processes, thereby affecting cell metabolism and apoptosis [274]. Functional enrichment analysis demonstrated that andrographolide targets the PI3K/Akt and HIF-1 signaling pathways to suppress the growth of GC [274]. Another study revealed that andrographolide inhibits MMP2/9 activity and downregulates anti-apoptotic Bcl-2 protein while upregulating tissue inhibitors of metalloproteinase (TIMP) and pro-apoptotic Bax protein, resulting in the prevention of GC cell proliferation, invasion, and metastasis [275]. Additionally, andrographolide was found to alter the expression of oncogenes such as survivin [275]. Moreover, in GC cells, andrographolide induced both non-apoptotic cell death and TNF-related apoptosis-inducing ligand (TRAIL)-mediated apoptosis by increasing ROS production and death receptor 5 expression [276].

### OC

In the context of OC, it was observed that ginkgolide B and WEB2086 exhibited inhibitory effects on tumor growth and reduced the proportion of CSCs [116]. This can be attributed to the ability of PAF to facilitate spheroids formation and impede the transition of quiescent OC cells into the active cell cycle via the PAF/PAFR signaling pathway [116]. Notably, in PAF-treated cells, there was a substantial increase in both the percentage of CSCs and the expression of genes associated with stemness [116]. According to another report, ginkgolide B demonstrates a wide range of cytostatic effects on OC cell lines, which include the suppression of cell proliferation and the initiation of apoptosis [277]. Moreover, rupatadine treatment has been shown to exert a notable anti-proliferative effect on OC cells, as evidenced by its ability to effectively impede in vitro proliferation and migration of various types of OC cells, including clear cell, serous, breast cancer gene 1 (BRCA1) mutant, and endometrioid cells [119].

### BC

The characteristics of CSCs in triple-negative BC (TNBC) are promoted by THO complex (THOC)-1, a component of the THOC, an evolutionally conserved RNA-binding protein complex that plays important roles in regulating mRNA elongation, termination, 3’-end processing, and export. Andrographolide treatment suppresses TNBC by reducing THOC1-promoted CSC characteristics [278]. Additionally, another study revealed that andrographolide exhibits anti-estrogenic properties by inhibiting proliferation and inducing apoptosis in estrogen-receptor-positive and progesterone-receptor positive (ER^+^/PR^+^) MCF-7 and triple-negative MDA-MB-231 BC cells [279]. These effects were mediated via the deactivation of the ER-α receptor and inhibition of the PI3K/AKT/mTOR signaling pathway [279].

Furthermore, andrographolide has been shown to induce apoptosis in p53 mutant, triple-negative MDA-MB-231 mammary epithelial carcinoma cells [280]. The antiproliferative activity of andrographolide was mediated through increased ROS generation, G2/M phase cell cycle arrest, mitochondrial membrane potential (MMP), and mitochondria-dependent caspase-mediated apoptosis involving caspase-9 and caspase-3 [280]. Andrographolide has demonstrated potential for treating BC bone metastasis [281]. Its anti-tumor activity, observed in both in vitro and in vivo studies, was mechanistically linked to the downregulation of the NF-κB signaling pathway and inhibition of MMP9 expression [281]. Moreover, kadsurenone has been suggested as a potentially effective approach to mitigate osteolytic bone metastases resulting from BC by inhibiting the PAF/PAFR signaling pathway [114].

### Bladder and prostate cancers

In this cancer model, ginkgolide B has been shown to block ZEB1 protein translation by upregulating miR-223-3p, which in turn decreases the invasiveness of bladder cancer cells [282]. Additionally, andrographolide-mediated inhibition of NF-κB and PI3K/Akt signaling pathways, both in vitro and in vivo, has been shown to reduce bladder cancer cell growth and increase apoptosis [283]. In prostate cancer, the anti-tumor mechanisms of andrographolide involve regulating genes associated with double-strand breaks, including ATM, NBN, BRCA2, BLM, PALB2, and BLM, leading to DNA damage induction [284]. Furthermore, it has been demonstrated that andrographolide can decrease prostate cancer cell growth by targeting the chemokine receptors CXCR3 and CXCR7, as well as cell cycle regulators [285].

### Glioblastoma

In a study, Othman et al. [286] investigated the efficacy of andrographolide as an anticancer agent and its potential molecular pathways in human glioblastoma multiforme (GBM). The findings indicated that andrographolide suppresses human brain cancer cell lines (DBTRG-05MG) by modulating the ERK1/2/c-Myc/p53 axis, leading to cell cycle arrest and subsequent activation of the apoptosis signaling pathway [286]. Similarly, cedrol exhibits anti-proliferative effects on GBM cells, as evidenced by its ability to reduce cell viability in a time- and dose-dependent manner [287]. Mechanistically, this effect was found to be achieved through enhanced ROS generation, activation of the DNA damage response, cell cycle arrest at the G0/G1 phase, and induction of apoptosis [287].

### Liver cancer

In hepatocellular carcinoma cells, ginkgolide C has been shown to exhibit anti-neoplastic properties by inhibiting cell proliferation and promoting apoptosis through the suppression of the hepatocyte growth factor (HGF)/c-Met signaling pathway [288]. Additionally, it was observed that ginkgolide C inhibits the invasion and migration of HepG2 cells, thereby blocking multiple key aspects of cancer growth [288]. In another study, the inhibitory effects of ADN-9, a 15-benzylidene substituted derivative of andrographolide, were examined on the growth and metastasis of murine hepatoma H22 cells in xenograft and orthotopic models [289]. The results demonstrated that ADN-9 significantly suppressed both orthotopic and subcutaneous xenograft tumors, leading to a remarkable reduction in tumor growth and metastasis [289]. Furthermore, treatment with 100 mg/kg ADN-9 (ig.) in orthotopic hepatoma-bearing mice resulted in a normalization of serum alpha-fetoprotein (AFP) levels, which is a crucial marker for liver carcinoma [289]. Compared to andrographolide, ADN-9 exhibited superior efficacy in reducing tumor size, inhibiting H22 cell invasion and metastasis, decreasing microvessel density, a marker of tumor angiogenesis, and induction of tumor cell apoptosis in a subcutaneous xenograft mouse model [289]. These findings were further supported by evidence indicating that the inhibitory effect of ADN-9 was linked to the attenuation of the VEGF/VEGFR2/AKT signaling pathway [289].

### Pancreatic cancer

The PAFR antagonist WEB2086 has been shown to inhibit pancreatic ductal adenocarcinoma (PDAC) tumor cell extravasation (metastasis) in wild-type mice, but exerted no effect in mice lacking CCAAT/enhancer-binding protein delta (C/EBPδ). This indicates that host C/EBPδ impacts tumor metastasis in a PAFR-dependent manner [290]. In another study, ginkgolide B treatment was demonstrated to inhibit the viability of BxPC-3, CAPAN1, PANC1, and Mia PaCa-2 pancreatic cancer cell lines in a dose-dependent manner [291]. While a low dose of ginkgolide B alone had no significant effect, when combined with gemcitabine, it augmented gemcitabine sensitivity, resulting in the suppression of cell proliferation and tumor growth as well as enhanced cell apoptosis via mechanisms involving the inhibition of the PAFR and NF-κB signaling pathways [291].

## Effects of PAF and PAFR antagonists in combination therapy efficacy

The development of tumor resistance remains a significant challenge in the therapeutic management of cancer patients receiving chemotherapy and/or radiotherapy [292]. This section will explore how antagonists targeting the PAF/PAFR pathway, when used in combination with radiotherapy and chemotherapy, may improve treatment efficacy.

### Radiotherapy

Radiotherapy is a crucial modality for treating various forms of cancer, which utilizes high-energy radiation to eliminate cancer cells and reduce tumor size [293, 294]. The therapeutic efficacy of radiation on tumors is determined by their sensitivity or resistance to radiation, which are key factors influencing treatment outcomes [295]. However, the frequent occurrence of radioresistance often hinders the effectiveness of radiation therapy and contributes to patient deterioration [296]. Studies in clinical radiobiology have identified four biological processes that influence cellular vulnerability to radiation: sublethal and potentially lethal damage repair, cell repopulation, cell cycle redistribution, and reoxygenation [297, 298]. A significant limitation of this treatment is the expedited proliferation of residual cells. Compensatory proliferation, an evolutionarily conserved process responsible for tissue regeneration in lower organisms, can also occur in tumor cells following cytotoxic radiation exposure [299]. Ionizing radiation (IR) induces the production of ROS, and multiple studies have shown that host immune response can be suppressed when exposed to different ROS-generating pro-oxidative stressors, a mechanism involving the lipid mediator PAF [157, 300–302]. Of note, these pro-oxidative stressors include aromatic hydrocarbons found in jet fuel, chemotherapeutic drugs, cigarette smoke, and ultraviolet B (UVB) radiation, which can directly affect glycerophosphocholines (GPC) and produce oxidized GPCs (ox-GPCs), potent agonists of the PAFR [303, 304].

IR can induce the production of PAF, leading to the activation of PAFR signaling, which hinders the effectiveness of radiotherapy [154]. Unlike the resistance mechanisms that occur within tumor cells, this process is likely mediated by the manipulation of the host’s immune response to the tumor [154]. Another study led by da Silva-Jr et al. [23] also validated that radiation exposure generates PAFR ligands, resulting in increased PAFR expression in tumor cells and contributing to tumor regrowth. Preventing the activation of PAFR with its potent antagonists such as CV3938 or PCA4280 before irradiation led to a further reduction in the survival of murine carcinoma cell line TC-1 cells [23]. This indicates that PAFR agonists play a critical role in protecting tumor cells from radiotherapy-induced cell death [23]. The study also noted that the inhibition of PAFR prevented the radiation-induced increase in TC-1 cell proliferation [23].

**Fig. 3:**
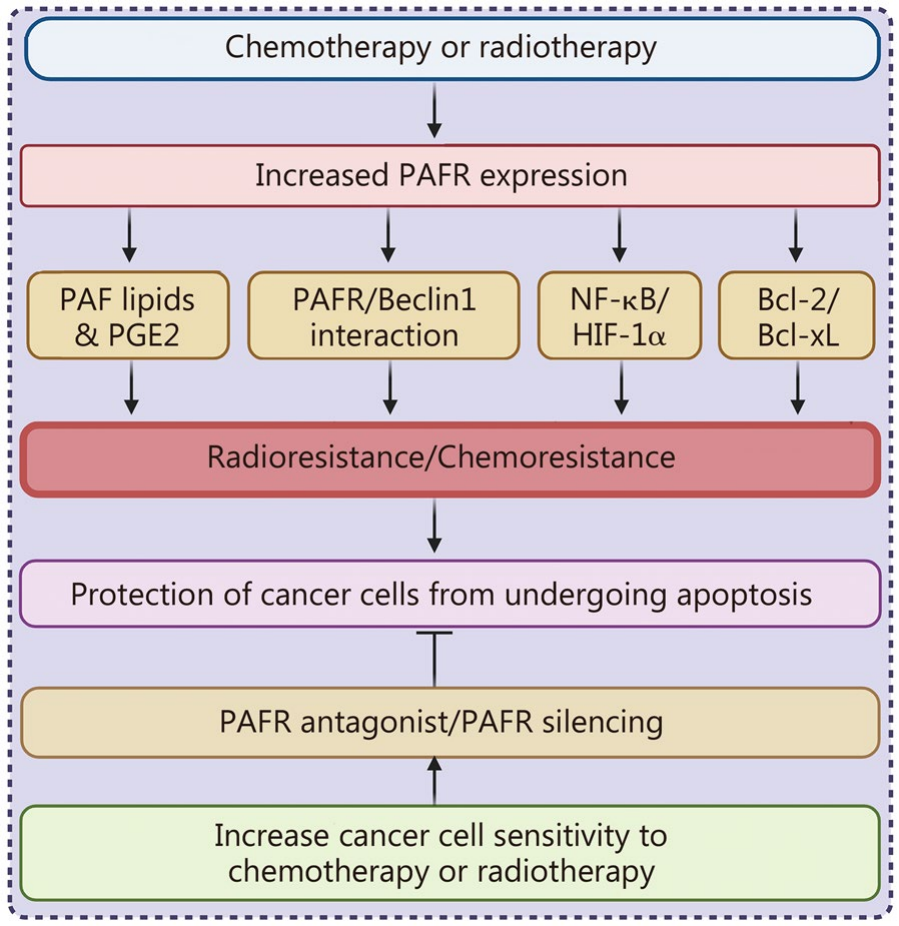
Mechanisms of platelet-activating factor (PAF)/platelet activating factor receptor (PAFR) signaling in radiotherapy and chemotherapy-induced effects. Radiotherapy or chemotherapy treatment to cancer cells increases PAFR expression, which augments the production of PAF lipids and PGE2, interacts with autophagy protein Beclin 1, and induces the downstream signaling cascades (e.g., NF-κB, HIF-1α), or upregulates anti-apoptotic proteins, Bcl-2 and Bcl-xL. These changes mediate radioresistance/chemoresistance, which protects cancer cells from undergoing apoptosis. PAFR antagonists/PAFR silencing block PAFR signaling and increase the sensitivity of cancer cells to radiotherapy and chemotherapy. Bcl-2 B-cell leukemia/lymphoma 2, NF-κB nuclear factor kappa-B, HIF-1α hypoxia-inducible factor 1α, PGE-2 prostaglandin E2

In line with these findings, da Silva-Junior et al. [24] identified that clinical samples of cervical cancer exhibit increased PAFR expression compared to normal cervical tissue. Additionally, exposure to radiotherapy augmented PAFR expression in cervical tumors [24]. Similarly, in vitro studies demonstrated that radiotherapy enhanced the expression of PAFR and triggered the production of prostaglandin E2 (PGE2) and PAF in cervical cancer cell lines [24]. These lipids activate the PAFR, thereby protecting tumor cells from radiotherapy-induced cell death. Importantly, treatment with PAFR antagonist CV3988 selectively increased the susceptibility of cervical cancer and squamous carcinoma cell lines to radiotherapy by inhibiting PAFR [24]. The schematic representation of signaling pathways involved in the development of resistance and approaches to enhance the efficacy of radiotherapy or chemotherapy is shown in Fig. 3.

In another report, Yao et al. [305] identified PAFR as the potential target through which radiation suppresses autophagy without activating the mTOR pathway. PAFR can bind to the essential autophagy protein Beclin 1, which results in the inhibition of its serine phosphorylation [305]. Notably, ginkgolide B, a PAFR antagonist, enhances the effectiveness of radiotherapy by interfering with the formation of the PAFR/Beclin 1 complex in PC3 and LNCaP cell lines, which exhibit increased levels of PAFR expression following the exposure to radiotherapy [305]. Significantly, ginkgolide B effectively increased the sensitivity of PC3 and LNCaP tumor xenografts to radiation, leading to a marked reduction in tumor size [305]. Overall, these findings suggest the potential of PAFR antagonists in improving the therapeutic outcomes for patients with prostate cancer undergoing radiotherapy [305].

### Chemotherapy

Chemotherapeutic agents are widely used to treat human malignancies by targeting multiple signaling cascades, including DNA replication and repair mechanisms [306]. Notably, the production of ROS is a common mechanism underlying chemotherapeutic agents-induced cell death [307–310]. However, various cellular resistance mechanisms have been identified that impact the effectiveness of chemotherapy in treating solid tumors, including melanoma [311–313]. In this context, systemic administration of the PAFR agonist CPAF has been shown to impede the anti-tumor efficacy of etoposide in an experimental melanoma model [314].

Similarly, a study conducted by Sahu et al. [153] demonstrated that the activation of PAFR signaling inhibits the efficacy of etoposide and melphalan chemotherapy in experimental melanoma models. Importantly, unlike chemotherapy resistance, which occurs at the level of tumor cells, this process was found to be mediated through host-PAFR signaling, which manipulates the immune response via immunosuppressive regulatory T cells (Tregs) [153]. The fact that chemotherapy induces the production of PAFR agonists in melanoma patients implies that the PAFR pathway may have significant clinical relevance. Of note, inhibition of COX-2, a downstream cascade of PAFR signaling, via systemic administration of pharmacological COX-2 inhibitors, enhanced the anti-tumor efficacy of chemotherapy by blocking Tregs-mediated immunosuppression [153]. Overall, these findings suggest that the PAFR/COX-2/Tregs axis could potentially be explored for melanoma intervention [153].

Another study by Seo et al. [315] discovered that PAF caused an upregulation in the levels of anti-apoptotic proteins Bcl-2 and Bcl-xL in B16F10 melanoma cells. Additionally, when tumor cells were exposed to both PAF and the chemotherapeutic drug etoposide, PAF protected melanoma cells from etoposide-induced cell death via activation of the NF-κB pathway. Similarly, Onuchic et al. [316] demonstrated that the administration of cisplatin to human melanoma cells (SKMel37) expressing PAFR resulted in an upregulation of PAFR in these cells. Exogenous PAF administration protected SKMel37 cells from the cytotoxic effects of cisplatin, preventing cell death. Furthermore, the combined administration of cisplatin and the PAFR antagonist WEB2086 significantly reduced the growth of SKMel37 tumor xenografts in nude mice [316]. These findings further support the premise that activation of the PAFR signaling can hinder the effectiveness of chemotherapy, and its inhibition via PAFR antagonists could be a promising approach to enhance chemotherapy efficacy.

In a model of OC, the chemotherapeutic drug cisplatin was found to induce the mRNA and protein expression of the PAFR [317]. This upregulation of PAFR expression was medicated by the activation of NF-κB and hypoxia-inducible factor 1α (HIF-1α) pathways [317]. Inhibition of PAFR using a siRNA approach or a PAFR antagonist resulted in an augmentation of cisplatin-induced cell death in human OC cells [317]. This suggests that PAF/PAFR may play a role in promoting tumor cell survival following genotoxic stress [317]. In a SKOV-3-luciferase xenograft model, researchers [317] demonstrated that the combined administration of cisplatin and the PAFR antagonist ginkgolide B effectively hindered tumor progression. These findings further support the notion that the PAF/PAFR axis plays a crucial role in tumor survival and that inhibiting this axis impedes tumor growth.

Kawasaki et al. [318] conducted a study on OSCC to examine the relationship between PAFR expression and sensitivity to cisplatin chemotherapy in seven OSCC-derived cell lines. They identified two cell lines (Ca9-22 and Ho-1-N-1) that were resistant to cisplatin and discovered that ginkgolide B, a selective inhibitor of PAFR, increased the susceptibility of these cell lines to cisplatin and promoted apoptosis. Subsequently, the researchers [318] assessed the downstream signaling pathways of PAFR in cells treated with PAFR-siRNA or ginkgolide B following cisplatin treatment. In both experimental settings, they observed a reduction in the phosphorylation of ERK and Akt, as well as an increase in the levels of cleaved caspase-3 [318]. These findings indicate that targeting PAFR via ginkgolide B could be a potential treatment strategy for modulating cisplatin sensitivity against OSCC.

Notably, Lou et al. [291] determined the impact of ginkgolide B on the sensitivity of gemcitabine in pancreatic cancer cell lines. The study demonstrated that ginkgolide B reduced the half maximum inhibitory concentration (IC50) of gemcitabine in a dose-dependent manner [291]. Additionally, ginkgolide B inhibited cell proliferation, enhanced cell apoptosis, and limited tumor growth when used in combination with gemcitabine [291]. However, ginkgolide B alone did not have any significant impact [291]. While gemcitabine alone induced PAFR expression and NF-κB/p65 phosphorylation, leading to an increase in NF-κB activity. This effect was largely inhibited when combined with ginkgolide B, [291]. Furthermore, ginkgolide B inhibited PAFR expression in a dose-dependent manner [291]. Knockdown of PAFR resulted in a substantial reduction in phosphorylated NF-κB/p65 levels, thereby inhibiting NF-κB activity and improving the sensitivity to gemcitabine, which induced cell death [291]. In summary, these findings indicate that ginkgolide B can enhance the sensitivity of pancreatic cancer cell lines to gemcitabine by inhibiting the PAFR/NF-κB pathway [291]. Additionally, the study suggests that ginkgolide B may offer therapeutic benefits when used in combination with gemcitabine for treating pancreatic cancer [291].

## Conclusions

While other published literature highlights the role and mechanisms of the PAF/PAFR pathway in cancer and cancer therapy, this review provides a comprehensive overview, including detailed insights into the effects and efficacy of natural and commercial PAF inhibitors and PAFR antagonists in various experimental cancer models. Importantly, as PAFR interacts with several tumor suppressor and oncogenic signaling cascades, targeting the PAFR axis represents a promising approach for cancer treatment. Overall, the studies utilizing PAF inhibitors and/or PAFR antagonists support the potential for their exploration in clinical settings. Notably, considering the ongoing challenges in cancer management with standard-of-care treatments such as chemotherapeutic agents and radiation therapy, including the development of tumor resistance mechanisms and adverse side effects, there is a critical need to explore strategies to overcome tumor resistance and enhance the therapeutic efficacy. Of significance, experimental studies have shown that targeting PAFR exerts synergistic effects and can improve the effectiveness of chemotherapy and radiation therapy. Given that increased PAFR expression in tumors has been correlated with disease progression, including enhanced growth, invasiveness, metastasis, and poor prognosis, the development of compounds with improved affinity and specificity should provide new insights into the PAF system and could result in novel targets and better outcomes for therapeutic agents.

## Data Availability

Not applicable.

## Abbreviations

BC: Breast cancer
CCT: Cytidine triphosphate (CTP): phosphocholine cytidyltransferase
COX-2: Cyclooxygenase type 2 enzyme
CPAF: Carbamyl-PAF
CSCs: Cancer stem cells
CTP: Cytidine triphosphate
EGF: Epidermal growth factor
ErbB: V-erb-b2 avian erythroblastic leukemia viral oncogene homolog 2
GC: Gastric cancer
GO: Gene ontology
GPC: Glycerophosphocholines
GPCR: G-protein-coupled membrane receptor
IL: Interleukin
IR: Ionizing radiation
KO: Knock out
LPCAT: Lysophosphatidylcholine acyltransferase
LPS: Lipopolysaccharides
MAPK: Mitogen-activated protein kinase
MMP: Matrix metalloproteinase
MMP: Mitochondrial membrane potential
MSCs: Mesenchymal stem cells
MyD88: Myeloid differentiation primary response protein 88
NF-κB: Nuclear factor kappa-B
NSCLC: Non-small cell lung cancer
OC: Osteosarcoma
OC: Ovarian cancer
OSCC: Oral squamous cell carcinoma
PAF: Platelet activating factor
PAFR: Platelet activating factor receptor
PC: Prostate cancer
PI3K: Phosphoinositide 3-kinase
RA: Rheumatoid arthritis
ROS: Reactive oxygen species
STAT3: Signal transducer and activator of transcription 3
THOC: THO complex
TLR4: Toll-like receptor 4
TNF-α: Tumor necrosis factor-α
UV: Ultraviolet
VEGF: Vascular endothelial growth factor
